# The Effects of Cardioprotective Antidiabetic Therapy on Microbiota in Patients with Type 2 Diabetes Mellitus—A Systematic Review

**DOI:** 10.3390/ijms24087184

**Published:** 2023-04-13

**Authors:** Ioana-Cristina Bica, Valeria-Anca Pietroșel, Teodor Salmen, Cosmina-Theodora Diaconu, Carmen Fierbinteanu Braticevici, Roxana-Adriana Stoica, Andra Iulia Suceveanu, Anca Pantea Stoian

**Affiliations:** 1 The Doctoral School, “Carol Davila” University of Medicine and Pharmacy, 37 Dionisie Lupu, 020021 Bucharest, Romania; ioana-cristina.bica@drd.umfcd.ro (I.-C.B.);; 2 Department of Diabetes, “Prof. Dr. N.C. Paulescu” National Institute of Diabetes, Nutrition and Metabolic Diseases, 030167 Bucharest, Romania; 3 The Department of Gastroenterology, The Emergency University Hospital Bucharest, 050098 Bucharest, Romania; 4 The Department of Diabetes, Nutrition and Metabolic Diseases, “Carol Davila” University of Medicine and Pharmacy, 050474 Bucharest, Romania; 5 The Gastroenterology Department, Ovidius University, 900527 Constanta, Romania

**Keywords:** microbiota, type 2 diabetes mellitus, GLP-1 RA, SGLT-2i, holistic approach

## Abstract

As the pathophysiologic mechanisms of type 2 diabetes mellitus (T2DM) are discovered, there is a switch from glucocentric to a more comprehensive, patient-centered management. The holistic approach considers the interlink between T2DM and its complications, finding the best therapies for minimizing the cardiovascular (CV) or renal risk and benefitting from the treatment‘s pleiotropic effects. Sodium-glucose cotransporter 2 inhibitors (SGLT-2i) and glucagon-like peptide-1 receptor agonists (GLP-1 RA) fit best in the holistic approach because of their effects in reducing the risk of CV events and obtaining better metabolic control. Additionally, research on the SGLT-2i and GLP-1 RA modification of gut microbiota is accumulating. The microbiota plays a significant role in the relation between diet and CV disease because some intestinal bacteria lead to an increase in short-chain fatty acids (SCFA) and consequent positive effects. Thus, our review aims to describe the relation between antidiabetic non-insulin therapy (SGLT-2i and GLP-1 RA) with CV-proven benefits and the gut microbiota in patients with T2DM. We identified five randomized clinical trials including dapagliflozin, empagliflozin, liraglutide, and loxenatide, with different results. There were differences between empagliflozin and metformin regarding the effects on microbiota despite similar glucose control in both study groups. One study demonstrated that liraglutide induced gut microbiota alterations in patients with T2DM treated initially with metformin, but another failed to detect any differences when the same molecule was compared with sitagliptin. The established CV and renal protection that the SGLT-2i and GLP-1 RA exert could be partly due to their action on gut microbiota. The individual and cumulative effects of antidiabetic drugs on gut microbiota need further research.

## 1. Introduction

Type 2 diabetes mellitus (T2DM) is a complex metabolic condition with an increasing incidence worldwide, with more than 500 million patients reported in 2021, according to the International Diabetes Federation (IDF) [1]. T2DM is a major cause of chronic kidney disease (CKD), blindness, and atherosclerotic cardiovascular disease (ASCVD) (which includes myocardial ischemia, a history of stroke, and lower limb arterial disease), significantly decreasing the quality of life in patients with hyperglycemia [2]. The Consensus Report by the American Diabetes Association (ADA) and the European Association for the Study of Diabetes (EASD), published in 2022, recommends a holistic patient-centered approach. The management of T2DM should include the treatment of hyperglycemia, weight control, and the assessment of cardiovascular risk (CV) factors, comorbidities, or complications, respectively [3]. The 2023 Standards of Care in Diabetes ADA guidelines advocate for GLP-1 RA or SGLT-2i use in patients with T2DM and ASCVD or CKD to reduce the risk of macro- and microvascular complications in this category of patients [4].

The Human Microbiome Project (HMP) investigates the microbial components of the human body and how different bacterial species influence health and predispose to various conditions [5]. In 2019, the Integrative HMP Research Network Consortium published results about the role of gut microbiota in inflammatory bowel diseases (IBD) and prediabetes [6].

Lately, the gut microbiota have been associated with different metabolic diseases, such as obesity [7] or T2DM [7,8], but also CV diseases (CVD) [9] and NAFLD [10]. In addition, dysbiosis, which includes various compositional and functional alterations of the microbiota, can be caused by genetic factors, infection, inflammation, diet, or some types of xenobiotics (drugs, food additives, antibiotics, or chlorinated water) [10].

Certain dysbioses have been described in modern conditions [11]. Researchers discovered that patients with obesity have an altered ratio between *Bacterioidetes* and *Firmicutes*, with a reduction in the first bacterial group and an elevation in the latter [12]. Moreover, patients with obesity who lost weight suffered from a change in the ratio between *Bacteroidetes* and *Firmicutes*, with improvement in favor of the first group [13]. Patients with T2DM and obesity have significantly higher amounts of *Bifidobacterium* spp. and fewer communities of *Lactobacillus acidophilus*, *Lactobacillus plantarum*, and *Lactobacillus reuteri* than controls [14]. Furthermore, a high-fat diet led to a decrease in *Bacteroidetes* and an increase in *Firmicutes* and *Proteobacteria*, independent of the nutritional status, highlighting the importance of diet in gut microbiota changes [15].

These changes in gut microbiota are described after analyzing the differences between groups using alpha- (within-sample) and beta-diversity (between-sample), respectively [16]. Alpha-diversity metrics are related to the richness, the distribution of abundances of the group, or both, indicating the variation within one sample [17]. Beta-diversity metrics determine which specimen is different from the other, indicating the variation between samples or treatment groups [18].

The microbiota have been demonstrated to have a significant role in CVD prevention through blood pressure control [19] or metabolic regulation, such as central appetite control [20], reducing inflammation [21], or altering the oxidation rate [22]. The composition and functional alterations in microbiota have been discovered to be linked to atherosclerosis. Bacteria from the *Collinsella* genus were correlated with symptomatic atherosclerosis in patients, while *Eubacterium* sp. and *Roseburia* sp. were increased/more prevalent in healthy individuals. Moreover, bacteria from the *Clostridiales* genus (*Clostridium* sp.) were associated with a high level of inflammatory C-reactive protein [23]. 

Trimethylamine N-oxide (TMAO) is a metabolite derived from the gut microbiome. It has been supposed to be involved in ASCVD in patients with diabetes and CKD because of the impaired renal excretion ability to eliminate it, especially in patients with advanced CKD (before hemodialysis or before a kidney transplant) [24]. Researchers discovered differences in the gut microbial composition in patients with diabetic kidney disease (DKD) compared to patients less prone to DKD (non-DKD), with higher levels of bacteria from the *Proteobacteria* phylum in patients with DKD [25]. The DKD presents a specific signature in terms of the bacterial (*Lactobacillus* sp., *Enterobacteriaceae*, *Ruminococus* sp., *Prevotella* sp., *Faecalibacterium* sp., *Clostridium coccoides*, *Clostridium leptum*) and fungal microbiome when compared to healthy control individuals [26].

Novel antidiabetic non-insulin drugs (SGLT-2i and GLP-1 RA) are promising for reducing CV events through various mechanisms [27,28,29,30,31,32]. In addition, one of the studied mechanisms in the pathogenesis of ASCVD is mediated through TMAO secreted by the gut microbiota [33]. This new hypothesis opens further therapeutic targets, thus emphasizing molecular research.

This review highlights current research on the effect of SGLT-2i and GLP-1 RA drugs on the gut microbiota as a risk factor for ASCVD. It also addresses approaches for targeting correlations between microbiota and other well-established CV risk factors.

## 2. Materials and Methods

**Figure 1:**
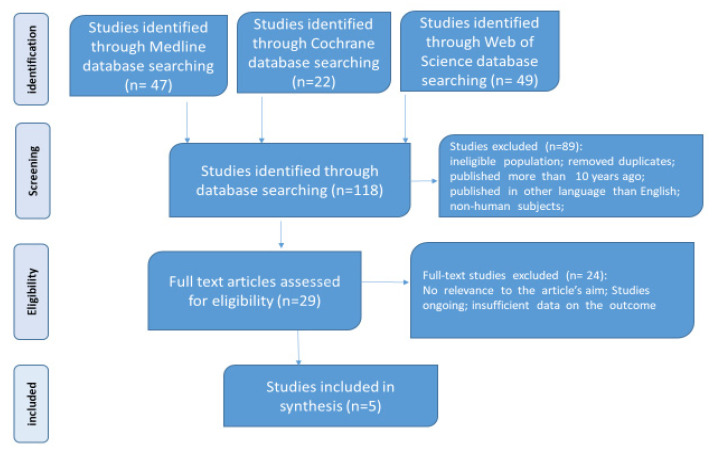
Flowchart for the study selection process according to PRISMA (Preferred Reporting Items for Systematic Reviews and Meta-Analyses) recommendations.

We developed a reproducible protocol for our study following the recommendations of Preferred Reporting Items for Systematic Reviews and Meta-Analyses (PRISMA) for the systematic review protocol checklist. Furthermore, we used the Population, Intervention, Comparison, Outcome, and Study (PICOS) Design strategy to guide the reasoning of our study and to carry out a clear, helpful, and systematic literature search. A systematic review of the literature was performed in January 2023 after CRD42022375026 PROSPERO’s registration number. We included only full-text articles, observational and randomized controlled trials, those on human adult populations (aged ≥ 18 years), those published in English, and those from the last ten years, with the following search criteria: “(type 2 diabetes mellitus OR diabetes OR diabetic) AND (microbiota OR microbiome) AND (SGLT-2i OR Empagliflozin OR Dapagliflozin OR Canagliflozin OR GLP-1 RA OR Dulaglutide OR Liraglutide OR Semaglutide OR Loxenatide)”. The inclusion criteria referred to patients diagnosed with T2DM who had received treatment with GLP-1 RA or SGLT-2i, in which microbiota were analyzed (using genomic DNA extraction and 16S rRNA gene amplicon sequencing). Forty-six articles were identified in the MEDLINE database, 71 in the Web of Science database, and 22 in the Cochrane database. Two researchers analyzed the titles and abstracts and selected five relevant articles. On the other hand, a third researcher intervened to resolve any problem in the selection process, as seen in Figure 1.

## 3. Results

### 3.1. The Gut–Cardio–Renal Axis

We did not identify randomized controlled trials (RCTs) that had, as a primary outcome, the effect of gut microbiota diversity or its production of metabolites (TMAO, SCFA) on ASCVD in humans. The effect can be inferred from the RCTs on the microbiota that have been reviewed in the following.

### 3.2. SGLT-2i Treatment and Gut Microbiota Interaction

In a double-blind, randomized controlled trial (RCT) that included 44 patients with T2DM, neither dapagliflozin nor gliclazide affected the microbiome diversity or composition after 12 weeks of treatment. Although gliflozins (-i) have different metabolic activities, there were no changes in the fecal microbiome in any treatment step. The methods and outcomes van Bommel et al. used are presented in Table 1. Using Bray–Curtis dissimilarity and multilevel principal component analysis, the authors detected a small shift in microbiome composition, but the compositional shift was not associated with treatment. However, dapagliflozin and gliclazide had opposite metabolic effects, the first leading to a reduction in fasting insulin levels, body mass index (BMI), fat mass percentage, and waist circumference [34]. 

Another randomized, open-label clinical trial investigated the effects of empagliflozin on the gut microbiota and plasma metabolites in patients with T2DM compared to metformin. Patients receiving empagliflozin had increased levels of sphingomyelin and reduced levels of glycochenodeoxycholate, cis-aconitate, and uric acid compared to metformin-treated patients. When analyzing Amplicon Sequence Variants (ASV), empagliflozin significantly improved (*p* < 0.05) the host’s microbiota by tilting the scale in favor of short-chain fatty acid (SCFA)-producing bacteria, such as *Eubacterium, Roseburia*, and *Faecalibacterium*, and lowering the levels of damaging bacteria, such as *Escherichia-Shigella*, *Bilophila*, and *Hungatella*, after a three-month treatment. There was a significant shift in the gut microbiome after 4 weeks of empagliflozin treatment (that remained relatively stable) in the Principal Coordinate Analysis (PCoA) of the Bray–Curtis distance [35].

### 3.3. GLP-1 RA Treatment and the Effect on Gut Microbiota

A 12-week, double-blind RCT by Smits et al. [36] investigated the effect of liraglutide and sitagliptin on the microbiota in patients with T2DM. They did not find any significant difference between liraglutide (1.8 mg subcutaneously weekly) and sitagliptin (100 mg daily) and the placebo in terms of alpha (*p* = 0.22) or beta (*p* = 0.23) microbiota diversity using PCoA. However, both liraglutide and sitagliptin increased fecal bile acid (deoxycholic acid, cholic acid, chenodeoxycholic acid, and ursodeoxycholic acid), in addition to lowering HbA1c by 1.3% (95% CI: −1.7 to −0.9) and by 0.8% (95% CI: −1.4 to −0.4), respectively. The methods and the number of patients are presented in Table 2 [36].

In contrast, another study demonstrated that liraglutide (1.2 mg subcutaneously weekly) induced gut microbiota alterations in patients with T2DM treated initially with metformin. As expected, after a 4-month treatment with liraglutide, the patients had lower HbA1c, BMI, and fasting and postprandial blood glucose levels and an improved lipid profile. In addition, researchers showed that a 4-month treatment with liraglutide positively correlated with specific microbial genera, especially *Firmicutes* and *Bacteroidetes.* The differences based on genomic characteristics were further investigated by LEfSe (Linear Discriminant analysis of Effect Size). Twenty-one species of bacteria were abundant in the pre-liraglutide-treatment group, and only 15 species were abundant in the post-liraglutide one. To compare the functional composition of the microbiome, KEGG (Kyoto Encyclopedia of Genes and Genomes) and COG (cluster of orthologous groups) were used. Three KEGG pathways (Glycolysis/Gluconeogenesis, G protein-coupled receptors, and Selenocompound metabolism) were enriched after treatment with liraglutide [37].

An 8-week trial investigated the effect of Polyethylene Glycol Loxenatide in one weekly injection in middle-aged and elderly patients with T2DM. Researchers assessed the intestinal flora at 0, 4, and 8 weeks in 12 patients with T2DM after one weekly injection of Polyethylene Glycol Loxenatide. After loxenatide, there was a trend of change in the number of genes, although it was not statistically significant (*p* > 0.05): an upward and then downward trend at the genus, species, and subspecies levels. Regarding microbial diversity, the overall trend was rising and then declining. In particular, at the genus level, there was one significant difference at baseline, week 4, and week 8—*Acinetobacter*—and at the species level—*Acinetobacter-unclassified*. The improvement in vascular endothelial function (determined by the tissue factor, plasminogen activator inhibitor-1, Endothelin-1, von Willebrand factor, tissue type fibrinogen activator, Prostaglandin I2 (PGI2), and LDL-cholesterol) was positively associated with *Bacteroides* and *Afebola* and negatively correlated with *Acinetobacter baumannii* [38].

## 4. Discussion

Several studies showed that T2DM led to endothelial dysfunction, reduced nitric oxide (NO) expression, and impaired vasodilation [39,40,41]. In addition, research demonstrated that the microbiota play a significant role in the relation between diet and ASCVD because some intestinal bacteria lead to an increase in SCFA and consequent positive CV effects through food breaking down/biodegradation [42].

### 4.1. Gut Microbiota-Mediated CV Risk in Type 2 Diabetes

Besides the mediator effect specified above, the microbiota could be used as a marker for CVD. Previous studies reported that the genera *Bacteroides* are present in the atherosclerotic plaque [43]. In the study of Tsai et al., published in 2021, the low abundance of *Bacteroides* was associated with left atrium enlargement and *Firmicutes* with a higher risk for left ventricular hypertrophy in T2DM subjects [41].

Still, there are several confounding factors in describing the relation between subclinical CVD and gut microbiota [41,44]. First, this relation varies over time, and the modifications of microbiota abundancy or diversity could have an accumulating effect [41]. Second, the number of patients included in studies is relatively small [41,42].

Third, it is difficult to separate the microbiota changes in T2DM described before [43,45,46] from those produced by other diseases. There is significant interest in the etiopathological role of microbiota in glycemic disturbances and insulin resistance. The mechanisms through which gut microbiota abnormalities lead to glycemic imbalance and T2DM rely mainly on the derived metabolites, the most widely known being SCFA (e.g., butyrate, propionate), bile acids, indoles, and lipopolysaccharide [45,46].

Finally, the microbiota co-evolves with the host and is influenced by the medication. The effects of antidiabetic drugs on dysbiosis have been reviewed without including the SGLT-2i class [47]. SCFA production is positively influenced by metformin and alpha glucosidase inhibitors [47]. The Consensus Report ADA-EASD 2022 recommends SGLT-2i or GLP-1 RA as first-line therapy, irrespective of the use of metformin, in addition to medical nutrition therapy (MNT) to reduce the cardio-renal risk in high-risk patients with T2DM [3]. In recent years, these two antidiabetic non-insulin classes have been studied for their beneficial impact on CV health [27,28,29,30,31,32], with increasing interest in their possible effects on the intestinal microbiota [34,35,36,37,38]. Additionally, the modulation of inflammation, including the possible mechanisms in SARS-CoV20-triggered cytokine response and the gut virome-bacterial microbiome interaction, needs further study [48].

### 4.2. Could the Cardioprotective Role of SGLT-2i Involve the Microbiota?

The SGLT-2i class inhibits glucose reabsorption in the proximal convoluted tubule of the nephron, leading to glycosuria and concomitant natriuresis [49]. In addition to its well-established effects on glucose control in patients with T2DM [4,50], multiple conditions have been demonstrated to be improved in patients treated with SGLT-2i agents, such as NAFLD [51,52], atherosclerotic cardiovascular disease [53], and chronic kidney disease [54], with a possible interaction of SGLT-2i on microbiota with cardio-renal and metabolic outcomes.

Dapagliflozin, empagliflozin, and canagliflozin revealed favorable effects on microbiota with tight interactions with T2DM, metabolic syndrome, and CVD in studies on mice [55,56,57,58]. Researchers used different rat categories, from diet-induced mice to subjects with nephropathy, and this heterogeneity overburdened the challenge of analyzing the impact of SGLT-2i on the microbiota. In other animal studies, the treatment with dapagliflozin has altered microbial diversity, especially Bacteroidetes, and Proteobacteria, in diabetic mice, with little effect on control animals (*p* < 0.05) [59]. Moreover, it was observed that the Firmicutes-to-Bacteroidetes ratio was significantly lower (*p* < 0.05) among diabetic mice than among the control group individuals [59]. However, the results from mouse trials cannot be extrapolated in human treatment decisions [60].

When we reviewed the studies in humans, contradictory results were found. Adding dapagliflozin to metformin did not lead to significant changes in the microbiota in patients with T2DM, as compared to the gliclazide treatment, suggesting that metabolic benefits in terms of glucose control, weight loss, and CV protection are not linked to the treatment’s interaction with the host’s microbiota [34]. Moreover, metformin itself exerts effects on the composition of microbiota [60,61]; therefore, dapagliflozin and gliclazide may overlap their benefits for gut microbiota with metformin ones. Metformin therapy may impact the gut microbiota through the improvement of the inflammatory status, gut permeability, and glucose metabolism or reshape the SCFA-producing bacteria population [47,61].

However, in a study by Deng et al., another SGLT-2i, empagliflozin, improved CV risk factors and glucose control, in correlation with an increase in SCFA-releasing bacteria, when compared to metformin therapy [35]. These differences between empagliflozin and metformin regarding the effects on microbiota occurred despite similar glucose control in both study groups, suggesting that the established CV and renal protection [25,62] empagliflozin exerts may be partly due to its action on gut microbiota.

Metformin plays an important role in interpreting the data researchers reported on the SGLT-2i effects on the microbiota. Contradictory results could be linked to metformin association or the comparison to new antidiabetic classes [34,35,36,37]. Dapagliflozin and liraglutide demonstrated better effects on gut microbiota in patients with T2DM only when these new agents were compared to metformin, but not in association with this first-line therapy, thus leading to new hypotheses about the cumulative beneficial effects of antidiabetic drugs on microbiota architecture. In addition, metformin has been described to induce changes in the bacterial species in the upper part of the small intestine that improve the SGLT1-dependent glucose sensing process in the upper part of the small intestine and thereby control glucose homeostasis [63].

### 4.3. GLP-1 RA Administration and the Impact on Gut Microbiota

GLP-1 RAs are potent antidiabetic drugs from the incretin mimetic class. Their hypoglycemic effect is based on increasing the pharmacologic level of GLP-1, which is altered in T2DM patients. Consequently, insulin secretion is stimulated in a glucose-dependent manner, while glucagon secretion is diminished. Furthermore, they possess anti-obesogenic properties, as they delay gastric emptying and promote satiety, hence decreasing alimentary intake. This effect may be explained by the aforementioned mechanism, as well as by the central mechanism [43,48]. GLP-1 RA directly interacts with receptors from the central nervous system, inducing the modulation of glutamatergic or GABA-ergic neurotransmission in brain cells, this mechanism being an explanation for their anorectic effect in patients with obesity and T2DM [64].

GLP-1 RAs possess pleiotropic actions. The protective CV effect resides in both direct anti-atherosclerotic effects as well as indirect mechanisms consisting of glucose control, weight reduction, lipid profile improvement, and, lastly, anti-inflammatory properties [14,48]. However, when investigating the specific role of microbiota in GLP-1 RA cardiovascular protective function, it was found that, when administering liraglutide to diabetic rats, it selectively enriched SCFAs-producing bacteria, such as *Bacteroides*, *Lachnospiraceae*, and *Bifidobacterium* [58].

The GLP-1 RA anti-inflammatory mechanisms are fairly complex and are still under investigation, as they involve various signaling pathways [65]. However, it is interesting to evaluate to what extent these immuno-modulatory properties are derived from either gut microbiota modification or a direct anti-inflammatory effect on the cardiovascular system [66]. Some microbiota dysbioses may alter the enteric nervous system, which may be the cause of GLP-1 resistance in patients with T2DM and a particular gut architecture signature [67]. GLP-1 has been demonstrated to decrease the hyperactivation of appetite- and reward-linked brain regions in patients with obesity, as compared to lean subjects [68].

Last but not least, liraglutide/GLP-1 RA may have beneficial effects on gut microbiota, preventing microbiome dysbiosis and endotoxemia, thus leading to a less severe form of coronavirus disease [48]. For the time being, we are still investigating the mechanisms by which GLP-1 RA agents promote beneficial metabolic effects, out of which the following are worth mentioning: glycemic control, weight loss, adipose tissue reduction, NAFLD improvement, and benefits on inflammatory status [69].

Liraglutide was studied for its impact on the gut microbiota in patients with T2DM, with contrasting results [36,37]. While Smits et al. [36] did not find any changes in alpha- or beta-diversity after 3 months, another 4-month treatment with a lower dose of liraglutide (1.8 mg vs. 1.2 mg subcutaneously daily) revealed positive effects on the microbial community, all correlated with better glucose control [37]. However, in the first case, the authors noticed that the lack of information and monitoring of the concomitant diet and associated medication may lead to different results [36]. Both studies admitted the reduced sample size, with an important effect on statistical significance. Moreover, none of them assessed the impact of diet composition and, therefore, diet change consequences on gut microbiota architecture. The concomitant use of metformin in the first study versus the switch from metformin to liraglutide in the latter may have an apparent role in the different results that both studies reported. The fact that metformin has been studied for its useful effects on the gut microbial composition should not be neglected [60,70].

Another argument for the positive effect of liraglutide on microbiota comes from a study on rodents. Liraglutide demonstrated a remarkable effect on obesity-related bacterial strains, decreasing the genera *Roseburia* and *Parabacteroides* (obesity-related) while increasing the genera *Blautia* and *Coprococcus* (weight loss-correlated) [71]. In another experiment on mice, the colonization of germ-free mice with gut microbiota from liraglutide-treated diabetic mice improved glucose-induced insulin secretion and regulated the intestinal immune system [72].

Another FDA-approved GLP-1 RA, loxenatide, demonstrated an impact on the intestinal microbial species, such as *Acinetobacter*, *Eubacterium,* and *Bacteroides,* in tight correlation with vascular endothelial function, metabolic control, and inflammatory status [38]. These GLP-1 RA cardio-metabolic protective effects may be based on their potential to inhibit the production of NO of TMAO and balance short-chain fatty acids (SCFA) production [73,74,75].

Kato et al. reported an interesting GLP-1 RA effect on the intestinal microbiota, with the activation of the sympathetic nervous system and consequent norepinephrine (NE) release in the intestinal lumen [76]. This mechanism explains the higher amount of *E. coli* in patients treated with GLP-1 RA, as reported in their study [76].

Incretin therapy has well-recognized gastrointestinal adverse effects (AE) [77], which may be involved in gut microbiota modulation in patients treated with GLP-1 RA. This new antidiabetic class can lead to nausea, vomiting, or diarrhea, with higher rates than for any other antihyperglycemic agents. These AE may be either one of the results of the gut microbiota-altering process or one of the possible pathways through which GLP-1 RA determines changes in the intestinal microbiota architecture. Future research is needed to clarify the link between GLP-1 RA tolerance/AE and their effects on gut microbiota.

### 4.4. Could Gut Microbiota Influence the GLP-1 RA Therapy Response?

While novel antidiabetic non-insulin therapies are studied for their potential effects on the gut microbiota, researchers focused on this relationship from the opposite point of view. Whereas there is tremendous interest in the GLP-1 RA effect on the human gut microbiota, some bacterial species have an important effect on GLP-1 RA metabolism. For example, gel-E, a secreted protease from *Enterococcus faecalis*, has been demonstrated to inhibit GLP-1 via direct cleavage [78]. This bacterial behavior would explain the difference in the efficacy of various antidiabetic classes. Moreover, this bi-directional relationship may have an impact on researchers in redirecting their attention toward the underestimated functions of the intestinal microbiome. Furthermore, acknowledging the relationship between microbiota and dietary and therapeutic molecules will help clinicians recommend an appropriate treatment plan for their T2DM patients.

Tsai et al. conducted a study that included patients treated with GLP-1 RA (liraglutide and dulaglutide), categorizing the host microbiota in responders to GLP-1 RA and in non-responders to therapy. They considered non-responder patients whose HbA1c and BMI did not significantly decrease after 12 weeks of GLP-1 RA treatment. While alpha-diversity did not differ among the responders and non-responders, as well as the *Bacteroidetes/Firmicutes* ratio (a hallmark of obesity), beta-diversity was the only significant marker. In one linear regression model, *Mitsuokella multacida*, *Bacteroides dorei, Lachnoclostridium* sp., and baseline HbA1c were significant predictors for an HbA1c decrease in T2DM patients treated with GLP-1 RA [71].

Besides treatment, associated complications influence the microbiota. He et al. demonstrated a different pattern regarding the intestinal microbiota in patients with DKD versus patients without DKD. *Shigella, Bilophila, Escherichia coli,* and *Bacteroides plebeius* were better represented in the DKD group, with *Citrobacter farmeri* and *Syntrophaceticus schinkii* being significantly and positively correlated with the urinary albumin creatinine ratio in the DKD group [62]. These results could give researchers and clinicians a better understanding of the underlying mechanisms of DKD. GLP-1 RAs are approved in DKD, and subsequent trials are needed to describe their effect on the microbiota of this category of vulnerable patients.

Our review is one of the few that investigate the effect of novel antidiabetic molecules, namely, GLP-1 RA and SGLT-2i, on the microbiota when administered to human subjects. However, this systematic review has encountered several limitations. The main drawback is the limited number of included studies and the small sample size, with important heterogeneity, as various antidiabetic molecules, different comparators, as well as diversely investigated and reported mediators, metabolic effects, and bacterial species were used. Taking all of this into account, it cannot be properly concluded if any of the mentioned effects are class-related. Furthermore, there is the probability that, in the case of the modification of the duration of treatment and the dose administered, other results may be achieved. Standardization is another important aspect that has to be considered, as the participants’ characteristics can also induce different baseline microbiota distributions. The differences between interspecies in terms of gut microbiota should not be overlooked, acknowledging the fact that the data from the studies on mice microbiota cannot be extrapolated to human subjects [56,57,58,59]. We have to consider the interracial differences in terms of the gut microbial architecture, because patients from different geographical areas may have different microbial signatures, partly because of genetic factors but also due to external factors, such as cultural patterns (different diet) or various environmental factors. Last but not least, it is extremely difficult to properly discriminate between the effects induced solely by the GLP-1 RA or the SGLT-2i on the microbiota and other confounding factors such as previous or concomitant antidiabetic treatment or simultaneous lifestyle modification (dietary, including sweetened beverages consumption, and physical activity), the latter being even harder to be quantified.

More comprehensive interventional studies are required to investigate the effect of novel antidiabetic molecules (GLP-1 RA and SGLT-2i) on microbiota, as well as the impact on metabolism and CVD from this perspective, and to consequently identify those actions as class effects or not.

## 5. Conclusions

The knowledge about the impact of therapeutic agents on microbiota in T2DM will improve the outcomes of patients, facilitate the choice between different types of therapies, and predict the response, but the high variability in microbial communities and the difficulties in identifying causality and in studying certain microbial taxa make this process arduous. The diversity of the gut–cardio–renal axis is only at the beginning of its description.

The SGLT-2i (empagliflozin) and GLP-1 RA (liraglutide and loxenatide) demonstrated beneficial effects on microbiota in patients with T2DM in terms of improving the colonies of *Eubacterium, Roseburia*, and *Faecalibacterium* and *Firmicutes* and *Bacteroidetes*, respectively, to the detriment of *Escherichia-Shigella*, *Bilophila*, *Hungatella*, and *Acinetobacter baumannii* in short-duration studies. However, liraglutide had contradictory results in a time- and dose-comparable RCT.

Subsequent investigations should include more details on SGLT-2i and GLP-1 RA activity regarding the intestinal flora and vice versa, with specific cardiovascular outcomes and longer follow-ups. Additionally, attention needs to be given to outcome standardization in future trials in order to properly compare homogenous results.

## Figures and Tables

**Table 1:** SGLT-2i effect on microbiota in T2DM.

Author	SGLT-2i	Control Group	Number of Patients (Study/Control Group)	Study Design and Duration	Method	Outcome
van Bommel et al. (2019) [34]	Dapagliflozin	Gliclazide	24/20	RCT 12 weeks	Fecal microbiota (Genomic DNA + 16S rRNA gene amplicon sequencing)	PCoA based on Bray–Curtis dissimilarity
Deng et al. (2022) [35]	Empagliflozin	Metformin	40/36	RCT 12 weeks	Gut microbiota (Genomic DNA + 16S rRNA gene amplicon sequencing) and plasma metabolites (LC-MS/MS)	Observed ASV Shannon index PCoA based on Bray–Curtis dissimilarity

DNA = Deoxyribonucleic Acid; 16 S rRNA = 16S ribosomal ribonucleic acid; LC-MS/MS = liquid chromatography–tandem mass spectrometry analysis; RCT = Randomized Clinical Trial; ASV = Amplicon Sequence Variants; PCoA = Principal Coordinate Analysis.

**Table 2:** GLP-1 RA effect on microbiota in T2DM.

Author	GLP-1 RA	Control Group	Number of Patients (Study/Control Group)	Study Design and Duration	Method	Outcome
Smits et al. (2021) [36]	Liraglutide	Sitagliptin/placebo	16/33	RCT 12 weeks	Fecal microbiota (16s rRNA amplicon sequencing) Fecal bile acids (ELISA)	Faith’s phylogenetic diversity (based on ASV) Shannon index PCoA based on Bray–Curtis dissimilarity
Shang et al. (2021) [37]	Liraglutide	Baseline/4 months	40	RCT 4 months	Fecal microbiota (16s rRNA amplicon sequencing)	ACE, Chao1, Shannon, and Simpson index PCoA based on Bray–Curtis dissimilarity KEGG, COG
Chen et al. (2022) [38]	Loxenatide	Baseline/8 weeks	12	RCT 8 weeks	Fecal microbiota (16s rRNA amplicon sequencing)	Gene number Shannon index PCoA based on Bray–Curtis dissimilarity Jensen–Shannon Divergence (JSD) distance-based beta diversity

16s rRNA = 16S ribosomal ribonucleic acid; ELISA = sandwich enzyme-linked immunosorbent assay; RCT = Randomized Clinical Trial; ASV = Amplicon Sequence Variants; ACE = Abundance-based Coverage Estimator; PCoA = Principal Coordinate Analysis; KEGG = Kyoto Encyclopedia of Genes and Genomes; COG = Cluster of Orthologous Groups.

## Data Availability

Not applicable.
